# Nanocrystalline ceria coatings on solid oxide fuel cell anodes: the role of organic surfactant pretreatments on coating microstructures and sulfur tolerance

**DOI:** 10.3762/bjnano.5.181

**Published:** 2014-10-06

**Authors:** Chieh-Chun Wu, Ling Tang, Mark R De Guire

**Affiliations:** 1 Department of Materials Science and Engineering, Case Western Reserve University, 10900 Euclid Avenue, Cleveland, Ohio, 44106-7204, USA

**Keywords:** cerium(IV) oxide, microstructure, organic self-assembled monolayers, solid oxide fuel cells, sulfur tolerance

## Abstract

Treatments with organic surfactants, followed by the deposition of nanocrystalline ceria coatings from aqueous solution, were applied to anodes of solid oxide fuel cells. The cells were then operated in hydrogen/nitrogen fuel streams with H_2_S contents ranging from 0 to 500 ppm. Two surfactant treatments were studied: immersion in dodecanethiol, and a multi-step conversion of a siloxy-anchored alkyl bromide to a sulfonate functionality. The ceria coatings deposited after the thiol pretreatment, and on anodes with no pretreatment, were continuous and uniform, with thicknesses of 60–170 nm and 100–140 nm, respectively, and those cells exhibited better lifetime performance and sulfur tolerance compared to cells with untreated anodes and anodes with ceria coatings deposited after the sulfonate pretreatment. Possible explanations for the effects of the treatments on the structure of the coatings, and for the effects of the coatings on the performance of the cells, are discussed.

## Introduction

Fuel cells convert chemical energy directly to electrical energy. Compared to conventional power sources, fuel cells offer higher efficiencies, lower emissions, modular installation scalable from milliwatts to megawatts, and distributed power generation to reduce transmission losses [1]. Among fuel cell technologies, solid oxide fuel cells (SOFCs) offer unique benefits [1–2]. They run not only on hydrogen, but also on widely available hydrocarbon fuels. They need little or no precious-metal catalysts. They provide high-quality utility-grade heat, which in combination with electrical efficiencies of up to 60% leads to total system efficiencies of 80–85%, exceeding conventional power sources. SOFCs thus have tremendous potential to meet rising global demand for electrical energy more efficiently and with lower environmental impact than conventional power sources.

A fuel cell consists of a dense ionically conducting layer (electrolyte) with porous electronically conducting layers (the electrodes) on each side, separating the fuel (e.g., H_2_) from its oxidant (typically O_2_ in air). In typical solid oxide fuel cells (SOFCs), oxygen molecules are reduced to oxide ions at the air electrode (the cathode) by electrons from the external circuit. The oxide ions cross the electrolyte and combine with H_2_ at the fuel electrode (the anode, the focus of the present study) to form H_2_O, releasing electrons into an external circuit to do electrical work before they pass to the cathode for consumption in the oxygen reduction reaction.

It is well known that the performance of SOFC anodes, typically composites of nickel metal with a zirconia or ceria ionic conductor, is degraded by sulfur impurities in the fuel, severely reducing both the power generated by the cell and its operating lifetime. (For recent reviews, see [3–4].) The extent and permanence of this “sulfur poisoning” varies with operating temperature, current density, sulfur concentration (as low as a few ppm), and anode materials [5–12]. Current consensus holds that adsorption of sulfur onto the nickel surface [13] may impede the ability of nickel to catalyze the oxidation of hydrogen [9,14–16]. Understanding sulfur poisoning is crucial to developing SOFCs that could operate on commercial, sulfur-containing hydrocarbon fuels (such as diesel and aeronautical fuels) and fuels derived from sulfur-containing sources such as coal.

Studies [17–21] have shown that incorporating ceria into the anode, either to replace yttria-stabilized zirconia (YSZ) as the ionic conductor or infiltrated into a porous anode structure, can lead to the reduction or elimination of sulfur poisoning. The procedures used by other groups to infiltrate ceria into SOFC anodes usually involve immersing the anodes into a precursor solution, e.g., of cerium nitrate [16,18,22–24] or through a sol–gel route [25]. After drying and high-temperature treatment, a ceramic film results.

Recent developments in the aqueous-phase deposition of functional oxides [26] can lead to a greater degree of control over the properties and morphology of films on SOFC anodes. Specifically, the surfaces of a commercial SOFC anode were treated with surfactants prior to immersion in an aqueous precursor solution [27]. By this approach, a nanocrystalline ceria film was formed without further heat treatment. The thickness of the film and its morphology and distribution within the microstructure of the porous SOFC anode depended significantly on the type of pretreatment used.

The present research sought to distinguish sulfur tolerance due to replacing YSZ with ceria from that due to protecting Ni from sulfur exposure. Few studies of sulfur poisoning have characterized the microstructural changes associated with the loss of performance [6,19–20,28]. In the present work the microstructural changes and the degree of sulfur tolerance were related to the presence or absence of the ceria coating, its morphology (which depended on the prior surfactant treatment), and the extent of sulfur exposure.

## Results

First we illustrate general characteristics of the performance of the cells in sulfur-containing environments. Then SEM and EDXS analyses of the microstructures of the cells, before and after operation, with and without surfactant pretreatments are presented. The performance of the cells, grouped by type of anode treatment, is then discussed to show correlations between surfactant treatment, coating characteristics, and cell performance. The anode treatments were of four types:

- Treatment 1: no ceria coating or surfactant treatment
- Treatment 2: ceria coating with no surfactant treatment (direct-treated)
- Treatment 3: ceria coating after thiol surfactant treatment
- Treatment 4: ceria coating after sulfonate surfactant treatment

### Effects of sulfur exposure on cell performance

The initial value of current density for each cell was chosen to give an output voltage of 0.7 V. If voltage dropped by more than 10% in a 24 h period, the current density was reduced to raise the voltage back to 0.7 V. Common measures of SOFC performance are the change in output voltage over time at a fixed current density, and area specific resistance (ASR, units of Ω·cm^2^).

**Figure 1:**
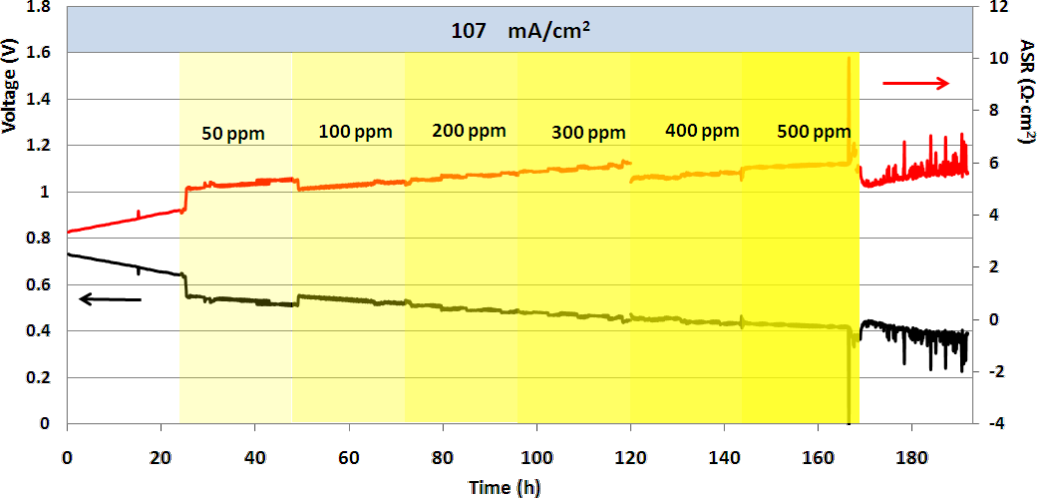
Output voltage and ASR at low current density, showing sulfur tolerance. Yellow shading denotes 24 h periods of H_2_S exposure in the anode stream at the concentration indicated. (Treatment-1 cell (no ceria coating) with no interlayer.)

Figure 1 shows the change in output voltage and ASR in a cell with no ceria coating (treatment 1) running on H_2_/N_2_ fuel at 107 mA·cm^−2^ (8.4% fuel utilization) throughout the 192 h test. This cell exhibited sulfur tolerance, i.e., only a gradual loss in power (no worse than that observed in sulfur-free fuel in the first 24 h of operation) throughout the test, though H_2_S levels progressively increased from 0 and 500 ppm in 24 h intervals. Such behavior was observed in many cells operated at current densities below 200 mA·cm^−2^, regardless of the presence or absence of a ceria coating.

**Figure 2:**
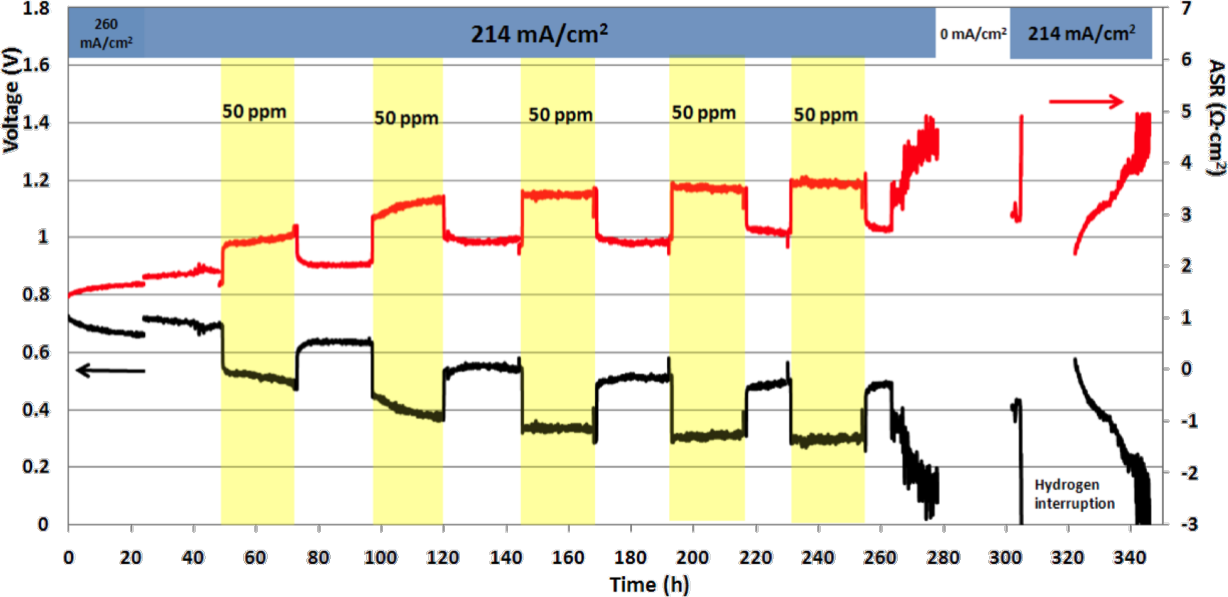
Output voltage and ASR, showing typical effects of partially reversible sulfur poisoning. Yellow bands denote periods of H_2_S exposure in the anode stream at the concentration indicated. (Treatment 3 cell, no interlayer.)

Figure 2 shows the change in output voltage and ASR in a treatment-3 cell operating while H_2_S levels alternated between 0 and 50 ppm in 24 h periods. The current density of 214 mA·cm^−2^ corresponded to fuel utilization of 16.7%. This test showed several hallmarks of sulfur poisoning [4–5,7,10]:

- a sharp initial drop in voltage on adding 50 ppm H_2_S to the fuel stream;
- a slower decrease in voltage on continued operation under constant atmosphere;
- recovery of most of the lost output voltage on reducing the H_2_S level to 0 ppm;
- overall decline in output voltage at constant current density, and a progressive rise in ASR (from 1.3 Ω·cm^2^ to 4.8 Ω·cm^2^) over the duration of the test.

This behavior was typical for many cells operated at current densities above about 200 mA·cm^−2^ [12], whether ceria-coated or not. In general, exposure to H_2_S led to shorter operating lifetimes and/or lower power (see Figure 12 below), resulting in lower total lifetime energy output.

**Figure 3:**
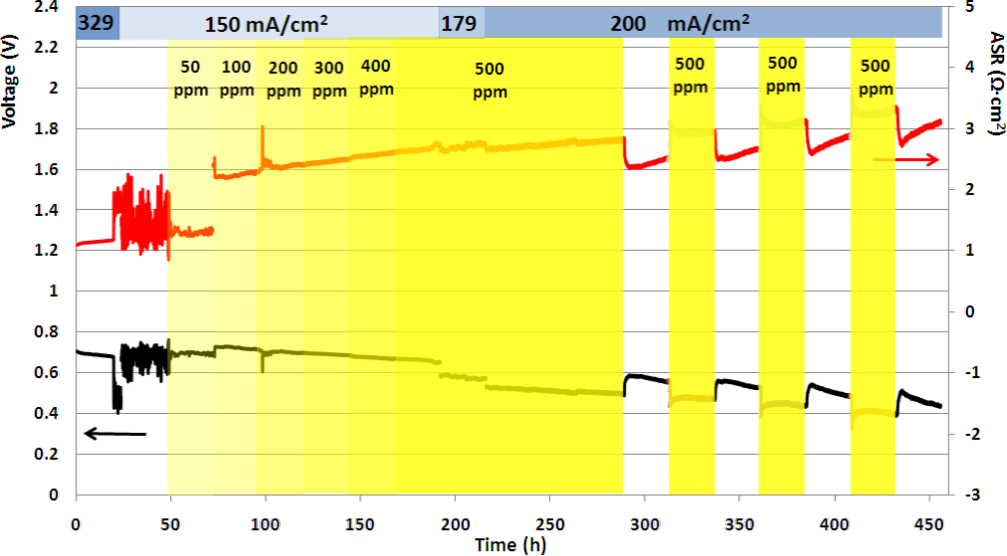
Output voltage and ASR, showing sulfur tolerance at a current density below 200 mA·cm^−2^ (24–192 h) and partially reversible sulfur poisoning at 200 mA·cm^−2^ (192–456 h). (Treatment-4 cell, no interlayer.)

Figure 3 underscores the significance of current density in the appearance of sulfur poisoning in the present study. It shows the change in output voltage and ASR in a treatment-4 cell that exhibited both sulfur tolerance at a current density of 150 mA·cm^−2^ (24–192 h) and partially reversible sulfur poisoning at 179–200 mA·cm^−2^ (192–456 h).

**Figure 4:**
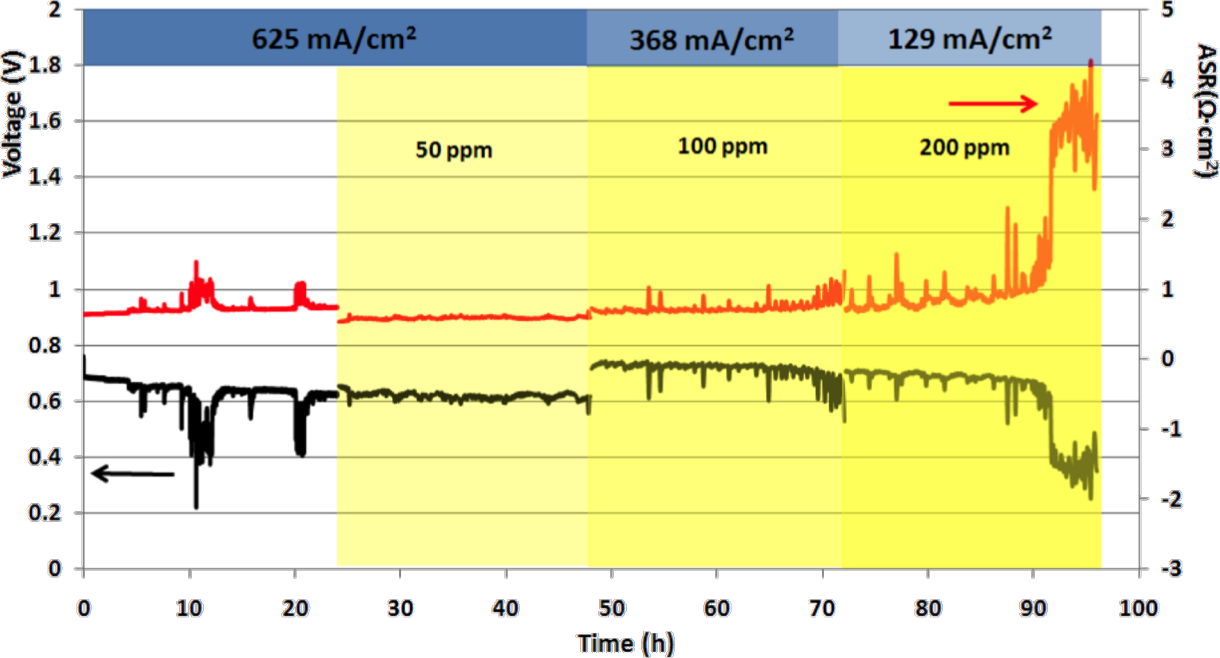
Output voltage and ASR, showing initial sulfur tolerance at high current density, and early cell failure. (Treatment-2 cell with GDC interlayer.)

Figure 4 shows the voltage output versus time of a treatment-2 cell that initially showed high sulfur tolerance, with little change in voltage or ASR on exposure to 50 ppm of H_2_S at high current density (625 mA·cm^−2^) for 24 h. Nevertheless, with each subsequent 24 h increase in H_2_S level (to 100 and 200 ppm) the current density had to be reduced sharply (to 368 and 129 mA·cm^−2^, respectively) to maintain the same voltage as at the preceding H_2_S level. (After about 90 h of testing, the rapid failure of the cell resulted from inadequate removal of H_2_O from the anode atmosphere [12].)

### Microstructures of as-treated anodes

The microstructures of coatings of cells with gadolinia-doped ceria (GDC) interlayers between the anode and electrolyte (see Experimental section for details) were essentially the same as those observed on cells without GDC interlayers. All SEM images shown here, except Figure 9, are of cells without GDC interlayers.

**Figure 5:**
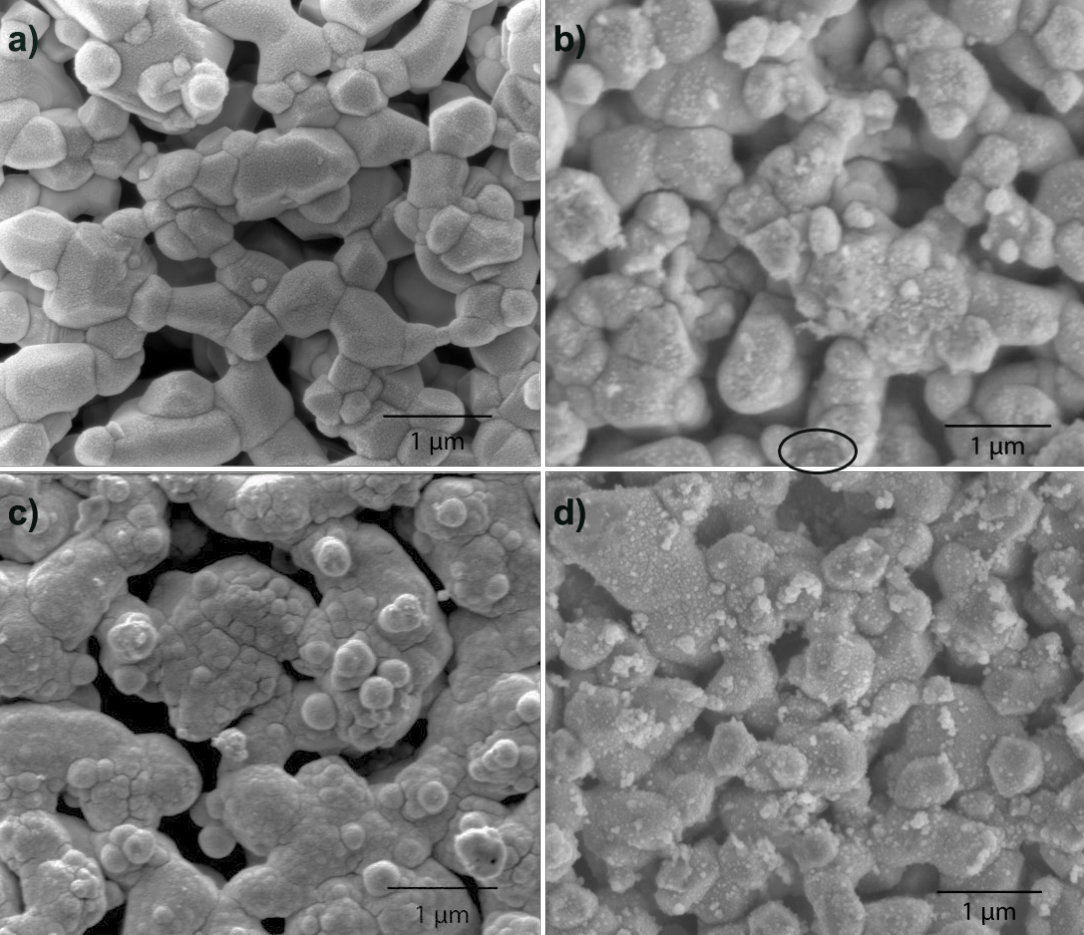
Top views of ceria deposition on NiO/GDC anodes. a) Treatment 1 (no coating). b) Treatment 2 (direct-treated). The ellipse indicates a gap in the coating. c) Treatment 3 (thiol-treated). d) Treatment 4 (sulfonate-treated).

The as-received anodes (i.e., before reduction of NiO to Ni) (Figure 5a) had a Ni:Ce atomic ratio of 3.47 (22.4 atom % Ce) (Table 1), in excellent agreement with the value of 3.43 computed from their nominal composition. (All reported Ni:Ce ratios and cerium concentrations were measured by using energy-dispersive X-ray spectroscopy (EDXS).) The NiO particles ranged in size from 0.5 to 1.5 μm and had faceted, polygonal faces (Figure 5a). The GDC particles were more rounded; many were sintered agglomerates ca. 3 μm long and ca. 1 μm wide.

Direct-treated ceria coatings (treatment 2) were mostly uniform and continuous (Figure 5b). The presence of a coating can be readily detected in the covering of the polygonal NiO grains, giving them a more rounded appearance. The untreated coating exhibited a few cracks at grain boundaries and occasional gaps (indicated by a circle in Figure 5b). The Ni:Ce atomic ratio was 2.60 (27.8 atom % Ce) (Table 1).

**Table 1:** Summary of typical EDXS analyses of Ce and Ni (Ni + Ce = 100 atom %) from Ni/GDC anodes (without GDC interlayer) in cells before and after testing, by type of pre-treatment. Data in the “after testing” columns were taken from the surfaces and cross-sections of the anodes shown in Figures 7 through 10.

treatment		before testing	after testing	
			surface	cross- section

1	Ni, atom %	77.6	69.4	69.2
	Ce, atom %	22.4	30.6	30.8
	Ni:Ce	3.47	2.27	2.25
	∆(Ni atom %)^a^	—	−8.2	−8.4
2	Ni, atom %	72.2	64.2	64.4
	Ce, atom %	27.8	35.8	35.6
	Ni:Ce	2.60	1.79	1.81
	∆(Ni atom %)^a^	—	−8.0	−7.8
3	Ni, atom %	66.8	65.3	67.5
	Ce, atom %	33.2	34.7	32.5
	Ni:Ce	2.01	1.88	2.08
	∆(Ni atom %)^a^	—	−1.5	0.7
4	Ni, atom %	76.6	76.8	69.2
	Ce, atom %	23.4	23.2	30.8
	Ni:Ce	3.27	3.30	2.25
	∆(Ni atom %)^a^	—	0.2	−7.4

^a^Change in atom % of nickel from start of testing to the end.

On thiol-treated anodes (treatment 3; Figure 5c) the ceria coating was uniform and continuous. Cracks in the coating were occasionally evident at the grain boundaries. The Ni:Ce atomic ratio was 2.01 (33.2 atom % Ce) (Table 1). On sulfonate-treated anodes (Figure 5d) the appearance of the anode was similar to that of the untreated anode, except that loose ceria clusters were evident. The Ni:Ce atomic ratio was 3.27 (23.4 atom % Ce) (Table 1).

**Figure 6:**
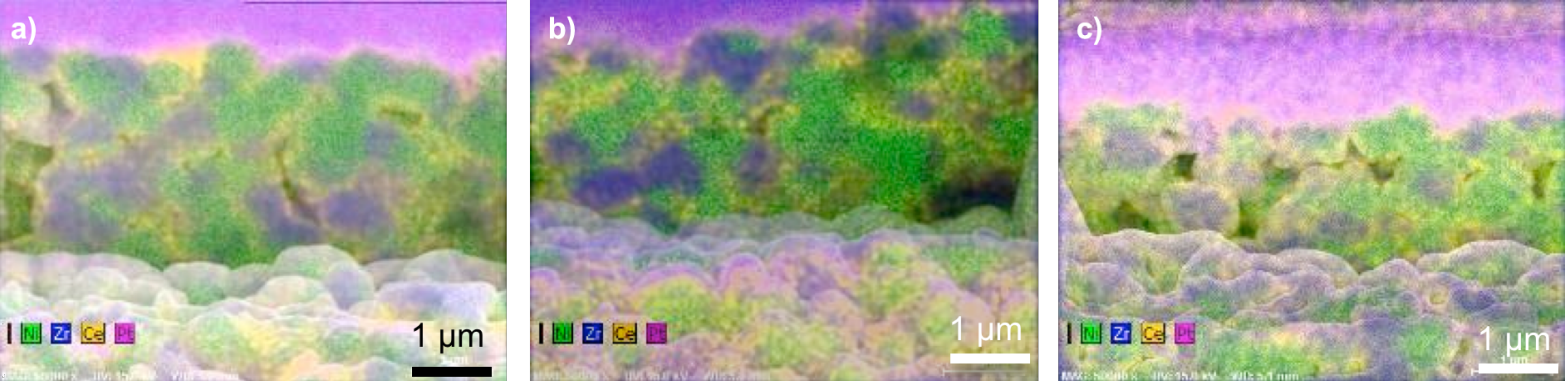
FIB cross-sections halfway through ceria-coated NiO/YSZ anodes, with superimposed EDXS maps (Ni: green; Zr: blue; Ce: yellow). a) Direct deposition (treatment 2). b) Deposition after thiol treatment (treatment 3). c) Deposition after sulfonate treatment (treatment 4). (Pink regions are the protective Pt layer applied as part of the FIB sectioning technique.)

Coating thicknesses were measured on cells with NiO/YSZ anodes that had been coated by using the same procedures as for the nickel-GDC anodes. Then cross-sections were prepared by using a focused ion beam unit, and EDXS maps were superimposed on the cross-sectional images (Figure 6). With YSZ replacing the GDC as the ionically conducting phase in the anode, the ceria coating could easily be distinguished. All three cross-sections showed ceria coatings enveloping both the NiO and YSZ grains. Figure 6 shows that the coating extended into the porous anode. The thicknesses of the coatings were determined from 5–10 locations in the underlying SEM images (not shown). Typical thickness values ranged from 60 to 170 nm on the direct-treated anode (Figure 6a), 100–140 nm on the thiol-treated anode (Figure 6b), and 50–110 nm on the sulfonate-treated anode (Figure 6c). Coating thicknesses typically varied in the order: thiol (treatment 3) > direct (treatment 2) > sulfonate (treatment 4). The ceria contents of these samples, as determined from overall EDXS analysis of the SEM images, decreased in the same order (Table 1, Ce atom % before testing).

### Microstructural analysis of anodes after operation

**Figure 7:**
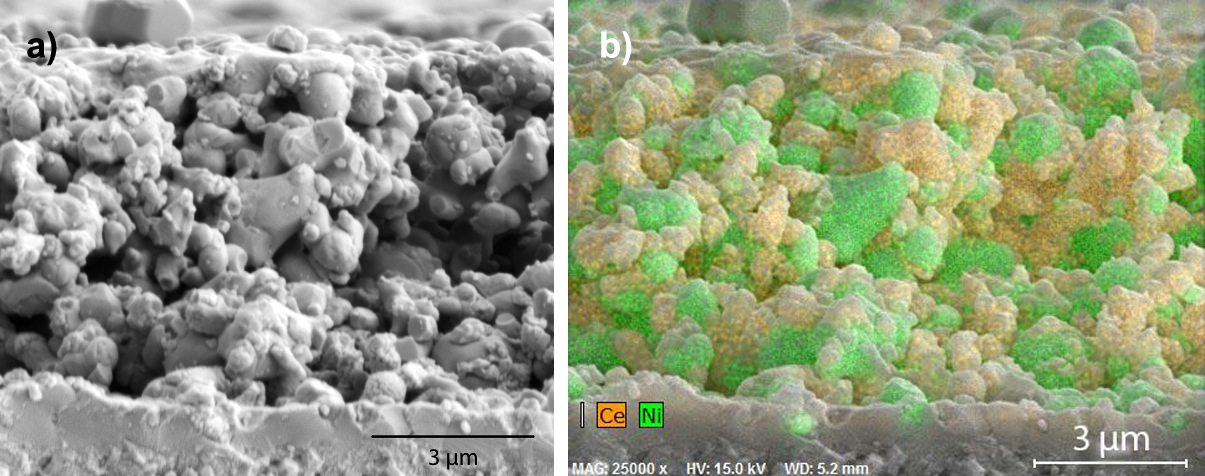
Cross-sectional view of an untreated Ni/GDC anode (treatment 1) after operation. a) SEM image; b) EDXS mapping of Ni (green) and Ce (yellow).

**As-received cells (treatment 1):** Figure 7 shows the post-operation cross-sectional SEM images of the anode of an as-received cell (i.e., no ceria coating). The cell was tested at an average current of 71 mA·cm^−2^ for 98 h with a total H_2_S exposure of 28.8 cm^3^. Even during this short test at low current density, some of the Ni particles had coarsened to over 2 μm in size (vs 0.5 to 1.5 μm before testing, Figure 5). At the top of the anode, coarsened Ni particles were spread on the surface. Both Ni particles and GDC particles were rounded without facets. The Ni:Ce atomic ratio was 2.27 at the surface and 2.25 at the cross-section, compared to 3.47 as received (Table 1). That is, Ni was depleted from the anode during operation, but had not preferentially segregated to the surface.

**Figure 8:**
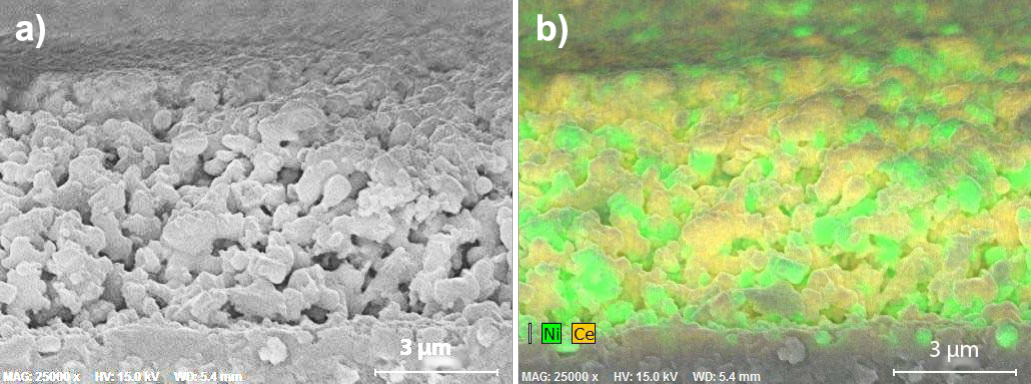
Cross-sectional view of a direct-treated anode (treatment 2) after cell operation. a) SEM image; b) EDXS mapping of Ni (green) and Ce (yellow).

**Direct-coated cells (treatment 2):** Figure 8 shows the anode of a direct-treated cell that gave an average current density of 135 mA·cm^−2^ for 109 h, with a total H_2_S exposure of 28.9 cm^3^. The testing conditions were comparable with the cell shown in Figure 7, but at nearly twice the current. The Ni:Ce atomic ratio was 1.79 at the surface, and 1.81 over the entire cross-section, compared to Ni:Ce = 2.60 at the surface of the coated anode before operation, i.e., depletion of Ni had occurred during operation, but Ni had not preferentially segregated to the surface.

**Figure 9:**
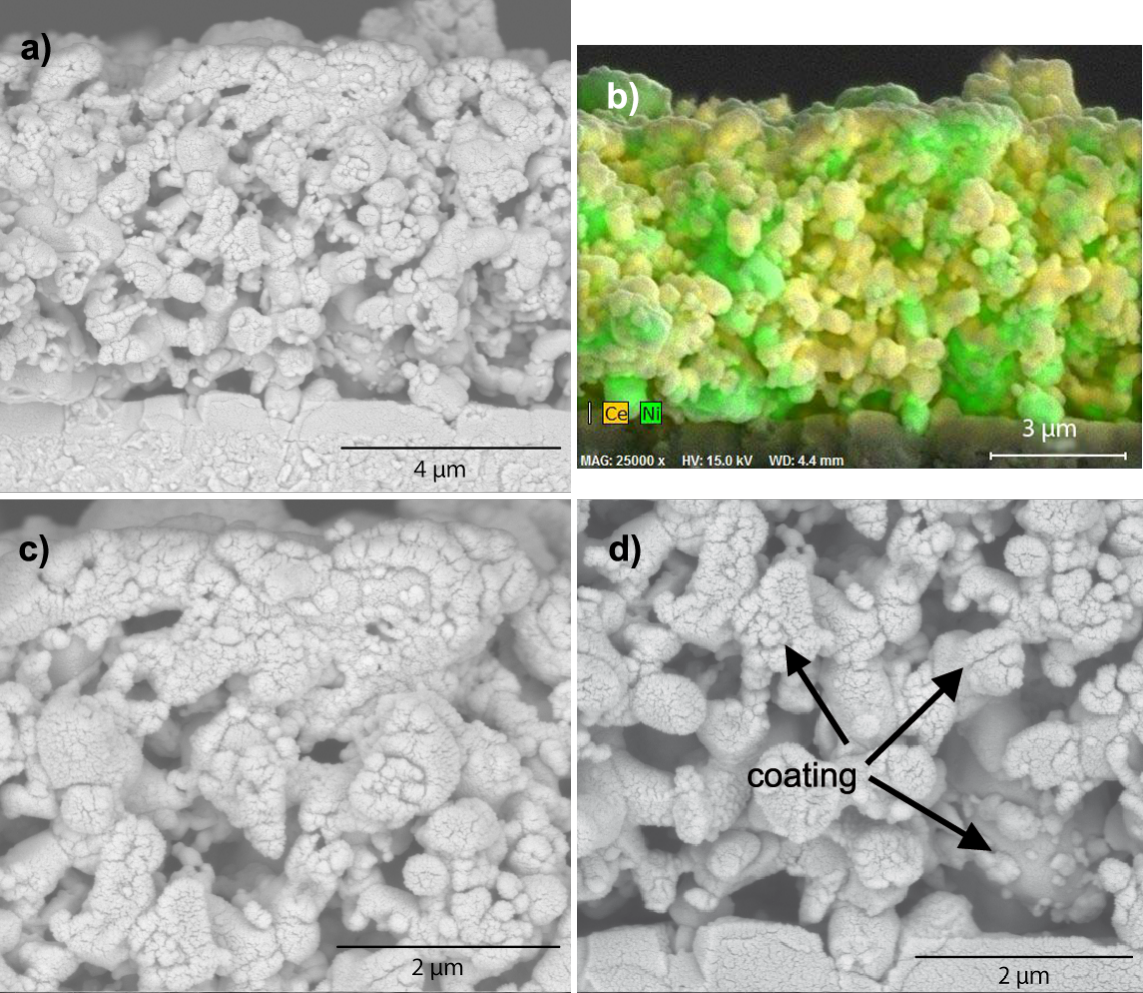
Cross-sectional views of the thiol-treated anode (treatment 3) of the cell shown in Figure 2 after operation. a) Whole anode thickness; b) EDXS mapping of Ni (green) and Ce (yellow); c) and d) higher magnification, c) near the anode surface and d) near the electrolyte (electrolyte is visible at bottom). Images c) and d) show that the ceria coating persisted throughout the anode. (Arrows in d point to coating edges or cracks).

**Thiol-treated cells (treatment 3):** Figure 9 shows the anode of the thiol-treated cell of Figure 2 after operation (average current density of 218 mA·cm^−2^ for 305 h of actual operation, with a total H_2_S exposure of 36 cm^3^). This test lasted nearly three times as long as that of the direct-treated cell (Figure 8). After operation, the remaining ceria film and film fragments could be observed at the anode surface and near the electrolyte (Figure 9). A few coarsened Ni particles over 2 µm in diameter, round with smooth surfaces, protruded from the anode surface. Pieces of the ceria film or of GDC particle fragments were observed on the coarsened Ni particles. The measured Ni:Ce ratio was 1.88 at the anode surface, and 2.08 over the cross-section (compared with 2.01 at the surface before operation). That is, a slight loss of Ni from the surface had occurred.

**Figure 10:**
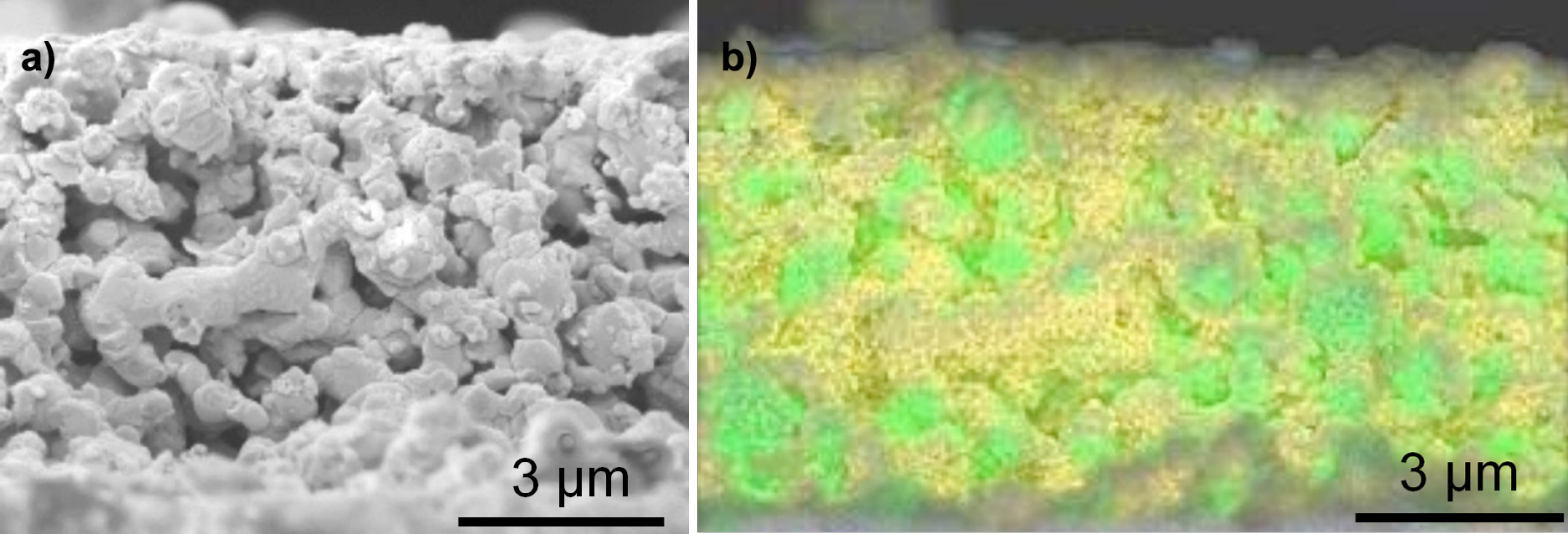
Cross-sectional view of a sulfonate-treated anode (treatment 4) after operation. a) SEM image; b) EDXS mapping of Ni (green) and Ce (yellow).

**Sulfonate-treated cells (treatment 4):** Figure 10 shows the anode of the sulfonate-treated cell of Figure 3 after testing (average current density of 187 mA·cm^−2^ for 456 h, with a total H_2_S exposure of 367 cm^3^). This was the longest test and the highest cumulative H_2_S exposure of the cells shown in Figures 1–4 and 7–10. Nickel and ceria phases were sintered into a porous two-phase network, with no signs of a ceria coating remaining. The measured Ni:Ce atomic ratio for the cross-section was 2.25, compared with 3.27 on the surface before operation, suggesting that nickel depletion from the interior had occurred during operation.

The loss of Ni from the anodes was especially noticeable for uncoated cells. Among the 17 anodes whose Ni distributions were analyzed after testing, on average, the trend for significance of this effect was: treatment 1 > treatment 4 ≈ treatment 2 > treatment 3. That is, the thicker the ceria coating, the less severe was loss of Ni from the anode.

### Overall cell performance

**Figure 11:**
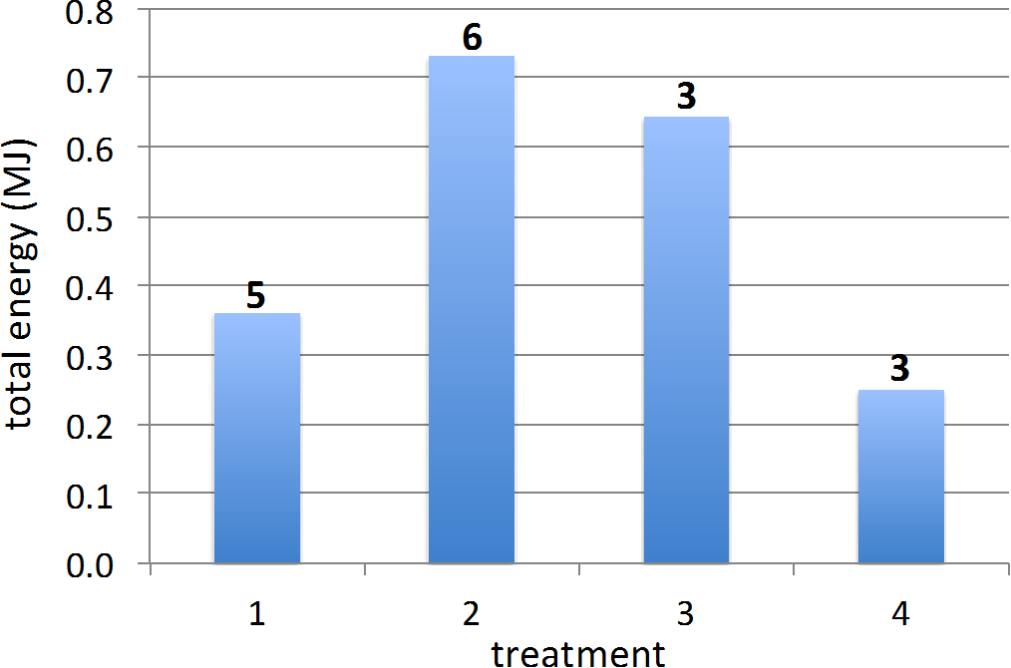
Average lifetime energy output of SOFCs (with and without GDC interlayers) tested in sulfur-containing fuel streams, grouped by treatment: 1, no coating; 2, direct ceria coating; 3, ceria coating after thiol treatment; 4, ceria coating after sulfonate treatment. The numeral above each column is the number of cells tested for each type of treatment.

Because the test protocol (see Experimental section) subjected the cells to a wide range of current densities and H_2_S exposures of various concentrations and durations (compare, e.g., Figures 1–4), and because of the different pre-treatments to which the cells were subjected, as well as performance variations between nominally identical cells, the cells exhibited significant variation in their operating lifetimes and output. As fuel cells are essentially energy-conversion devices, one useful metric for assessing the relative performance of devices that differed not only in their anode structures, but also in the details of their operating history, is total electrical energy output over the life of the device. Figure 11 shows the average total energy output of the cells (with and without GDC interlayers), grouped by anode treatment, in tests entailing H_2_S exposure. On average, the direct-treated and thiol-treated cells provided 103% and 78.5% more energy over their lifetimes than did the untreated cells, whereas the sulfonate-treated cells provided 31% less energy than the untreated cells.

**Figure 12:**
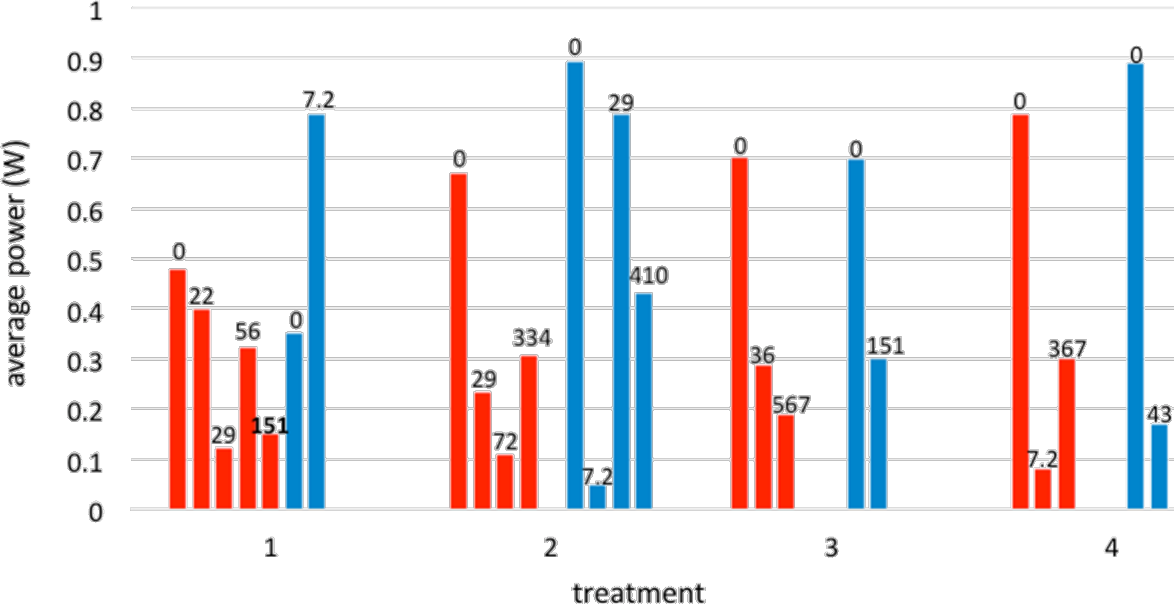
Average power over cell lifetime, grouped by anode treatment and anode type, ranked by cumulative H_2_S exposure within each group. Numbers at the top of each column indicate the cumulative H_2_S exposure over the lifetime of each cell. Blue columns: cells with GDC interlayers between anode and electrolyte. Red columns: cells without GDC interlayers.

Plotting the average power over the lifetime of individual cells versus total H_2_S exposure for the four types of treatment (Figure 12, which includes all of the cells averaged in Figure 11, plus cells that underwent no H_2_S exposure) gives another perspective on the effectiveness of the ceria coatings at improving sulfur tolerance. For cells with the same treatment, the average power mostly decreased with increasing cumulative sulfur exposure over the cell lifetime, but only ceria-coated cells (treatments 2, 3, and 4) survived total H_2_S exposure greater than 120 cm^3^. Figure 12 also indicates that for cells that experienced no H_2_S exposure, all the ceria-coated cells exhibited higher average power than the uncoated cells (treatment 1). This suggests that the ceria coating, regardless of the details of the pre-treatment, improved the average power output of the cells over their lifetimes.

## Discussion

The microstructures of the anodes changed greatly during operation and depended strongly on the testing conditions. The most notable changes occurred in the nickel phase: coarsening (in almost all cases), and nickel depletion from the interior in most anodes. The effects of the coatings and of the surfactant treatments on these phenomena are discussed below.

### Dependence of coating characteristics on surfactant treatments

Whatever effects the organic surfactants had on the cell performance could have only been exerted during the deposition of the ceria coatings. Before cell operation, during the high-temperature reduction of NiO to Ni (see Experimental section), the surfactants were undoubtedly burnt out, as similar surfactant layers have been shown to pyrolyze below 400 °C, even in low-oxygen atmospheres [29].

During deposition, the solution parameters (concentration, temperature, and pH) can be expected mainly to dictate the particle size and ultimate crystalline form of the coating [30]. The effects of the surfactant layer will be seen primarily in the extent to which it promoted the attachment of the solid particles from the deposition medium and affected their distribution on the substrate (in this case, a NiO–GDC composite).

In previous studies of oxide film deposition on surfactant-treated surfaces, sulfonate surfaces strongly favored the formation of continuous films of ZrO_2_, TiO_2_, and SnO_2_ [26]. This outcome is attributed to the high negative surface charge density of well-packed sulfonate surfaces under the acidic conditions at which these oxides precipitate from solution [31]. When the same treatments that we described here were used prior to applying ceria coatings to a different SOFC anode design than that used in the current work [27], the sulfonate surface gave the thickest and most continuous coatings.

In the present study, the fact that the sulfonate treatment gave the thinnest and least uniform ceria coatings can be attributed to the nature of the deposited surfactant layer. X-ray photoelectron spectroscopy (XPS) of the sulfonate-treated anodes (before ceria deposition) showed carbon and sulfur signals much higher than expected [32] from, e.g., a well-packed surfactant monolayer, and many times higher than the signals detected in the sulfonate-treated anodes of [27]. This indicates that the sulfonate treatment on the present anodes left large oligomers and cross-linked clusters of surfactant, which could have obscured or neutralized most of the sulfonate functionality and led to the thinner, less uniform ceria coatings.

Conversely, XPS measurements of the thiol-treated anodes before ceria deposition showed that most of the thiol functionality had oxidized to sulfonate. So it appears in the present work that the thiol surfactant provided a surface more like a well-packed sulfonate layer than did the sulfonate treatment (Figures 5c vs 5d; Figures 6b vs 6c), resulting in thicker and more uniform ceria coatings from the thiol treatment than from the sulfonate treatment.

### Relation of cell performance to coating characteristics and surfactant treatments

The effects of the surfactant treatments on the coatings are reflected in the sulfur tolerance of the variously treated anodes (Figure 11 and Figure 12). In light of the current thinking that sulfur blocks catalytic sites for the anode reaction on the nickel, the ceria coating may act to impede the sulfur adsorption on the nickel while still allowing the anode reaction to proceed. The current results then suggest that a continuous coating (direct- or thiol-treated), but not necessarily the thickest (thiol-treated) provides best sulfur tolerance to the anode. Aspects of the sulfonate treatment, particularly the oxone oxidation step, may have adversely affected the anode surface chemistry. (As explained in the Experimental section, we observed chemical damage to the cathode if the oxone solution contacted it, and subsequently took steps to prevent such contact before testing began.)

#### Nickel coarsening

Coarsening of nickel is often associated with performance degradation in cermet SOFC anodes [3,16,33–34]. One significant effect of the ceria coating in the current work was to hinder nickel coarsening. For example, compare the significantly coarsened nickel in Figure 7 (an untreated cell) to the nickel in Figure 8 (an direct-treated cell), tested for a similar time but at twice the current and power. Similarly, compare Figure 7b to Figure 9b and Figure 10b (thiol- and sulfonate-treated cells), which showed similar coarsening though the coated cells experienced many times longer and more intensive operation. Coarsening of the metallic phase reduces the density of three-phase boundaries between pore, electronic conductor, and ionic conductor, which are essential to cell operation [35]. Coarsening of Ni also leads to less interconnection of metal particles and therefore to a decrease of the electrical conductivity of the anode. This would be expected to result in loss of power over time, e.g., increased ASR.

The ceria coatings could not suppress nickel coarsening indefinitely. The coatings were readily observed after early stages of cell operation, but in the anodes of long-lived cells the ceria coating was visible mainly as small fragments on coarsened nickel particles. Most of the coating had presumably sintered into, and was indistinguishable from, the GDC phase of the anode. This suggests that eventually the ability of the coating to hinder coarsening broke down, allowing coarsening to proceed until the cell failed.

#### Nickel depletion

Lussier et al. [8] reported Ni depletion from Ni/YSZ and Ni/GDC anodes during operation in sulfur-containing atmospheres. Likewise in the present work, by comparing the anode composition before and after cell operation, loss of nickel from the anode was detected for most cells, especially uncoated cells and cells running at high current for long times [32,36]. This suggests that the ceria coatings hindered Ni depletion. The mechanism of nickel depletion is believed to involve the formation of low-melting, volatile Ni(OH)_2_ in the presence of the H_2_O formed at the anode [37]. A ceria shell around the metal network may act as a physical barrier to impede Ni(OH)_2_ formation or evaporation, with the most continuous coatings being most effective.

## Conclusion

Overall, this work established that nanocrystalline ceria coatings could be deposited throughout porous cermet anodes of SOFCs 6 µm thick by using an aqueous infiltration technique at 50 °C in 48 h without subsequent heat treatment. The morphology of the coatings – specifically, their thickness and their continuity – could be affected through surfactant pretreatments. Lastly, continuous uniform coatings 60–170 nm thick, as deposited directly on the anodes without prior surfactant treatment, or 100–140 nm thick as deposited on thiol-treated anodes, significantly improveed the sulfur tolerance in the tested cells.

The improvements in sulfur tolerance in ceria-coated anodes were attributed to the ability of the coatings to suppress Ni coarsening and depletion in the anode, and this effect was most pronounced in the thiol-treated and the direct-treated cells. The protective effect of the ceria coating appeared to diminish in cells where the coating had not remained physically intact and continuous.

## Experimental

The cells used in this study were electrolyte-supported, circular “button” cells, 3.8 cm in diameter. The electrolyte was Y_0.03_Zr_0.97_O_2−δ_, 100 µm thick. The anode, 6 µm thick, consisted of 60 wt % NiO and 40 wt % Gd_0.1_Ce_0.9_O_2−δ_ (gadolinia-doped ceria, GDC). The NiO was reduced to Ni during the initial heat-up of the cell under a flowing H_2_/N_2_ stream before cell operation began.

Some anodes (the cell of Figure 4, cells included in the averages in Figure 11, and cells represented by blue columns in Figure 12) in addition contained a 2 µm-thick, porous GDC interlayer between anode and electrolyte. This interlayer had no discernible effect on the characteristics of the ceria coatings, either before or after testing, which are the focus of the current work; the conclusions presented here apply equally to both of these types of anodes.

The cathode, 12 µm thick, was composed of 50 wt % Y_0.08_Zr_0.92_O_2-δ_ and 50 wt % lanthanum strontium manganite with La:Sr ratio of 0.85:0.15. The area of each electrode was 2.8 cm^2^.

### Surfactant treatment and ceria deposition

All cells were first cleaned with ethanol and dried in flowing argon. For the thiol treatment, the cleaned substrates were immersed in 1-dodecanethiol (CH_3_C_11_H_22_SH) for 5 h at room temperature in air, then washed in flowing ethanol for 2 min and in deionized water for 2 min.

For the sulfonate treatment, the cleaned cells were immersed in 1 vol % 1-bromo-11-(trichlorosilyl)undecane (Cl_3_SiC_11_H_22_Br) in bicyclohexyl (C_12_H_22_) for 5 h at room temperature in air. The trichlorosilyl groups hydrolyze and undergo condensation reactions with oxide and hydroxide groups on the electrode surfaces, and with each other. The intended result is a siloxy-anchored, bromine-terminated, cross-linked organic monolayer on the pore walls of the electrode. Then the specimens were refluxed with 7% potassium thioacetate in ethanol at 80 °C for 16 h to replace the –Br end groups with thioacetate (-SCOCH_3_). Lastly, the thioacetate was converted to sulfonate (-SO_3_H) by exposing the anode to saturated oxone (2KHSO_5_·KHSO_4_·K_2_SO_4_) aqueous solution for 2.5 h at room temperature [31]. During this step, instead of immersing the cell into the oxone solution, a cotton pad saturated with oxone was used to cover only the anode side of the cell. This technique provides enough oxone to oxidize the thioacetate group to sulfonate, while preventing damage to the cathode by reaction with the oxone.

Prior to ceria deposition, the surfactant depositions were monitored with X-ray photoelectron spectroscopy (XPS, PHI Model 5600 MultiTechnique System), with particular attention to the presence of the characteristic functional groups and to the progress of the in situ transformations entailed by the sulfonate treatment.

To deposit the ceria coating, the cells (with or without surfactant pre-treatment) were immersed in an aqueous solution of 0.01 M cerium acetate and 0.005 M potassium chlorate [38] at 50 °C for 48 h. After the deposition, the cells were rinsed with deionized water then ethanol and dried with argon.

### Cell operation

Current collectors, consisting of a Pt wire (Alfa Aesar, 0.30 mm dia., 99.9% metals basis) spot-welded to Pt mesh (Alfa Aesar, 52 mesh, woven from 0.1 mm-diameter wire, 99.9% metals basis), were bonded to both electrodes with Pt-based ink (Heraeus). The cell was then sealed to the end of a 3.2 cm-diameter stabilized zirconia tube (by using a silicate-based paste fired at 1050 °C for 1 h) with the anode facing an alumina gas feed tube inside the zirconia tube. This assembly was then put into a vertical tube furnace for cell operation.

Cell operation was conducted at 900 °C. The cathode side of the cell was exposed to the air atmosphere of the furnace. Fuel of 25 sccm H_2_ and 25 sccm N_2_, humidified to 3% water vapor by passing through a water bubbler at room temperature, was fed to the anode through the alumina tube. For each tested cell, open circuit voltage (OCV) was checked at the start of operation and every 24 h thereafter to be between 1.0 and 1.1 V, and testing was ended if the OCV was below 1.0 V. Cells were operated galvanostatically (by using an Autolab electrochemical analyzer or an Amrel electronic load with a power supply) at a current that produced a cell voltage of 0.7 V. If the cell voltage dropped more than 5% in 24 h, the current was reduced until the voltage reached 0.7 V. If the cell voltage dropped less than 5% in the first 24 h, H_2_S was introduced to the fuel stream at 50 ppm for an additional 24 h. If the cell voltage dropped by less than 5% during this period, the level of H_2_S was raised to 100 ppm, 200 ppm, 300 ppm, 400 ppm, or 500 ppm in successive 24 h periods. If the voltage dropped by more than 5% during any of these stages, the cell was run in the original sulfur-free gas flow for 24 h. Testing was ended if the voltage dropped to 0.4 V or if the current density had dropped to <50 mA/cm^2^ after a long decline in performance. After operation, the cells were cooled in flowing humidified H_2_/N_2_ to near room temperature. The current collectors were carefully peeled from the electrodes before analysis.

### Cell characterization

Scanning electron microscope (SEM) images of the anodes were taken (FEI xT Nova Nanolab) at 5 kV accelerating voltage. Energy-dispersive X-ray spectroscopy (EDXS) mapping at beam energy of 15 keV, combined with SEM images, was used to resolve the phases of anode particles qualitatively and to investigate the microstructure changes. The chemical compositions of the anode were analyzed (QUANTAX Esprit 1.8 software) on EDXS maps taken over an area of 190 μm^2^ or greater. Cross-sectional images through the coated anodes were obtained by using the focused ion beam unit of the Nova Nanolab.
